# Supplementary material for: In Vitro Effect of the Common Culinary Herb Winter Savory (Satureja montana) against the Infamous Food Pathogen Campylobacter jejuni
Source: Foods. 2020 Apr 24;9(4):537. doi: 10.3390/foods9040537 (PMC7230815; doi:10.3390/foods9040537)
Supplement: Supplementary file 1 [file foods-09-00537-s001.pdf]

Supplementary information

Table S1. Strain designation, description and reference of strains used in the study.

C. jejuni strain designation	Description	Reference
11168	NCTC reference strain	NCTC
Δ*cmeB*	Insertional mutant in the gene *cmeB* from *C. jejuni* NCTC 11168	[19]
Δ*cmeR*	Insertional mutant in the gene *cmeR* from *C. jejuni* NCTC 11168	[19]
Δ*cmeF*	Insertional mutant in the gene *cmeF* from *C. jejuni* NCTC 11168	[19]
Δ*cmeG*	Insertional mutant in the gene *cmeG* from *C. jejuni* NCTC 11168	This study; [6]

Table S2. UHPLC-PDA-ESI-MS analysis of the ethanolic extract of *Satureja montana* herbal parts.

Peak	[M+H]+	[M-H]-	UV	Compound
1	361	359	330, 290sh	Rosmarinic acid
2	539	537	325, 299	Caffeoyl rosmarinic acid isomer
3	593	591	335, 270	Apigeninmethyl ether hexosyl-deoxyhexosiderhamnoside
4	361	359	347, 291, 256	Trihydroxy-trimethoxyflavone isomer
5	315	313	337, 277	Dihydroxy-dimethoxyflavone isomer
6	164, 165*	164, 163*	264sh, 257	Thymoquinone
7	---**	---**	278, 219, 199	Carvacrol

*low intensity; in mixture with a dihydroxy-trimethoxyflavone isomer

**no signalionization in ESI positive and negative mode

Table S3. Chemical analysis of *S. montana* essential oil

Peak	Ret. Time	RI	Relative proportion (area %)	Substance
1	5~~,~~.616	925	0~~,~~.333	α-Thujene
2	5~~,~~.806	932	0~~,~~.391	α-Pinene
3	6~~,~~.212	947	0~~,~~.195	Camphene
4	7~~,~~.039	977	1~~,~~.06	1-octen-3-ol
5	7~~,~~.423	990	0~~,~~.269	Myrcene
6	8~~,~~.286	1016	0~~,~~.704	δ-2-Carene/δ-3-Carene
7	8~~,~~.601	1025	19~~,~~.25	*p*-Cymene
8	8~~,~~.715	1028	0~~,~~.315	Limonene
9	8~~,~~.796	1030	0~~,~~.465	1,8-Cineole
10	9~~,~~.794	1057	3~~,~~.274	γ-Terpinene
11	10~~,~~.091	1066	0~~,~~.874	(not identified)
12	11~~,~~.28	1098	0~~,~~.355	(not identified)
13	11~~,~~.36	1100	0~~,~~.935	δ-3-Carene
14	13~~,~~.158	1144	0~~,~~.186	Camphor
15	14~~,~~.02	1164	0~~,~~.963	Isoborneol
16	14~~,~~.518	1176	0~~,~~.767	Terpinen-4-ol
17	14~~,~~.856	1185	0~~,~~.39	(not identified)
18	15~~,~~.092	1190	0~~,~~.232	Isosylvestrene
19	17~~,~~.44	1246	0~~,~~.213	Carvone
20	17~~,~~.638	1250	1~~,~~.812	Thymoquinone
21	19~~,~~.116	1285	0~~,~~.202	trans-Anethole
22	19~~,~~.486	1294	1~~,~~.447	Thymol
23	20~~,~~.066	1307	63~~,~~.402	Carvacrol
24	20~~,~~.63	1321	0~~,~~.398	2,5-Diethylphenol
25	24~~,~~.692	1418	0~~,~~.271	Caryophyllene (E-)
26	28~~,~~.335	1509	0~~,~~.246	β-Bisabolene
27	30~~,~~.994	1577	0~~,~~.327	Isolongifolene
28	31~~,~~.137	1581	0~~,~~.725	β-Longipinene
